# Toll-like receptors in cellular subsets of human tonsil T cells: altered expression during recurrent tonsillitis

**DOI:** 10.1186/1465-9921-7-36

**Published:** 2006-02-27

**Authors:** Anne Mansson, Mikael Adner, Lars Olaf Cardell

**Affiliations:** 1 Laboratory of Clinical and Experimental Allergy Research, Department of Otorhinolaryngology, Malmö University Hospital, Lund University, 205 02 Malmö, Sweden

## Abstract

**Background:**

The palatine tonsils have a pivotal role in immunological detection of airborne and ingested antigens like bacteria and viruses. They have recently been demonstrated to express Toll-like receptors (TLRs), known to recognize molecular structures on such microbes and activate innate immune responses. Their activation might also provide a link between innate and adaptive immunity. In the present study, the expression profile of TLR1-TLR10 was characterized in human tonsil T cells, focusing on differences between subsets of CD4^+ ^T helper (Th) cells and CD8^+ ^cytotoxic T lymphocytes (CTL). The study was also designed to compare the TLR expression in T cells from patients with recurrent tonsillitis and tonsillar hyperplasia.

**Methods:**

Tonsils were obtained from children undergoing tonsillectomy, and classified according to the clinical diagnoses and the outcome of tonsillar core culture tests. Two groups were defined; recurrently infected tonsils and hyperplastic tonsils that served as controls. Subsets of T cells were isolated using magnetic beads. The expression of TLR transcripts in purified cells was assessed using quantitative real-time RT-PCR. The corresponding protein expression was investigated using flow cytometry and immunohistochemistry.

**Results:**

T cells expressed a broad repertoire of TLRs, in which TLR1, TLR2, TLR5, TLR9 and TLR10 predominated. Also, a differential expression of TLRs in CD4^+ ^and CD8^+ ^T cells was obtained. TLR1 and TLR9 mRNA was expressed to a greater extent in CD4^+ ^cells, whereas expression of TLR3 mRNA and protein and TLR4 protein was higher in CD8^+ ^cells. CD8^+ ^cells from infected tonsils expressed higher levels of TLR2, TLR3 and TLR5 compared to control. In contrast, CD4^+ ^cells exhibited a down-regulated TLR9 as a consequence of infection.

**Conclusion:**

The present study demonstrates the presence of a broad repertoire of TLRs in T cells, a differential expression in CD4^+ ^and CD8^+ ^cells, along with infection-dependent alterations in TLR expression. Collectively, these results support the idea that TLRs are of importance to adaptive immune cells. It might be that TLRs have a direct role in adaptive immune reactions against infections. Thus, further functional studies of the relevance of TLR stimulation on T cells will be of importance.

## Background

Tonsillar diseases are among the most common health-related problems in the general population, and tonsillectomy is one of the most frequently performed surgical procedures in the western world. The two main indications for tonsillectomy are recurrent tonsillitis and tonsillar hypertrophy [1]. The pathogenesis of recurrent tonsillitis is largely unknown [2], though it has generally been regarded as an infection caused by *Streptococcus pyogenes*, in particular group A β-hemolytic streptococci (GAS), or viruses [3,4]. It is still debated whether *Haemophilus influenzae *(HI) and *Staphylococcus aureus *(SA), other common bacteria that harbor in the deep crypts of the tonsils [2,4], play a role for the recurrent disease.

The palatine tonsils are secondary lymphoid organs located at a critical position for immunological detection of airborne and ingested antigens [5]. They are sites where innate immunity leads to onset of the adaptive immunity, mediated by B and T lymphocytes [6]. Naïve lymphocytes circulate between the blood and the secondary lymphoid organs in search of antigens. Once antigens are encountered within a secondary lymphoid organ, the passing naïve B and T lymphocytes bearing specific antigen receptors are retained [7]. After being activated, they undergo clonal expansion and after a few days acquire effector functions and immunological memory [6-9]. In contrast, the innate immune response provides protection immediately after an infectious challenge [10]. Members of the Toll-like receptor (TLR) family have emerged as central in this defence, through their ability to recognize conserved molecular structures on pathogens that are not present in higher eukaryotes, so called pathogen-associated molecular patterns (PAMPs) [10]. To date, 10 human TLRs (TLR1-TLR10) have been identified [11], all of which are transmembrane proteins with an extracellular leucine-rich domain and a conserved cytoplasmic domain. Each TLR recognizes specific PAMPs [12], including bacterial lipoproteins and lipoteichoic acids (TLR2), double-stranded viral RNA (dsRNA; TLR3), lipopolysaccharides (LPS; TLR4), flagellin (TLR5), imidazoquinolines and single-stranded viral RNA (ssRNA; TLR7 and TLR8) and unmethylated CpG-DNA (TLR9) [13-17]. TLR1 and TLR6 only signal as a dimer when combined with TLR2 [18], and the ligand for TLR10 is yet unknown [10]. Recognition of PAMPs by TLRs triggers a signaling pathway that leads to activation of nuclear factor κB (NF-κB) transcription factors and members of the MAP kinase family [19,20]. This activation does not only initiate innate immune responses, it also triggers adaptive immunity via induction of cytokines, chemokines and co-stimulatory molecules [5]. In addition, Gelman and colleagues [21] have reported that mouse CD4^+ ^T cells expressing TLR3 and TLR9 respond to CpG-DNA and poly(I:C) (ligand for TLR3) stimulation with NF-κB activation. This observation suggests that infectious organisms via TLRs can directly activate the adaptive immune response, which raises questions regarding the role of TLRs in T cell activation both with and without antigen-presenting cells (APCs).

Expression of TLRs has been detected in various upper airway tissues such as tonsils and adenoids, as well as in different cells of the immune system, e.g. monocytes, macrophages, dendritic cells (DCs) and B cells [5]. However, little is known about the expression in human T cells. The expression of TLRs in lymphocytes other than B cells has been a controversial issue and further information is needed for the understanding of the link between TLRs and adaptive immunity. In the present study, we are first to investigate the expression pattern of TLR1-TLR10 in cellular subsets of human tonsil T cells at both mRNA and protein level. The ability of TLRs to recognize PAMPs and to activate pro-inflammatory mechanisms might be of great importance for immune reactions in the tonsils. To address this issue, we compared the TLR expression *ex vivo *in T cells from recurrently infected and hyperplastic tonsils.

## Methods

### Patients

Tonsils were obtained from 39 children undergoing tonsillectomy under general anaesthesia at Malmö University Hospital (Malmö, Sweden). The study was approved by the Ethics Committee of Lund University and written informed consent was obtained. After the tonsillectomy, swabs were taken for tonsillar core cultures (representing the microbial flora of the tonsillar crypts) in order to determine the presence of pathogenic β-hemolytic streptococci and anaerobes. The patients providing the tonsils were divided into two groups, referred to as infected or control, based on their clinical diagnosis and the outcome of the core culture (Table 1). The infected group consisted of 18 patients referred to tonsillectomy because of multiple episodes (at least four times during the year preceding the surgery) of GAS tonsillitis (small to substantially enlarged tonsils, positive cultivation test). The control group consisted of 21 patients who underwent tonsillectomy because of tonsillar hyperplasia (no history of recurrent tonsillitis, negative cultivation test). None of the patients displayed symptoms of acute infection at the time of surgery, and none of them had received any antibiotic treatment for at least one month prior to surgery. Apart from the tonsillar symptoms, all patients were healthy and did not receive any medication.

**Table 1:** Demographic data of the patients

	Infected group	Control group
Number of patients	18	21
Sex	8 males, 10 females	8 males, 13 females
Age (years)	3–19 (mean 9.4, median 9)	3–18 (mean 8.2, median 6)
Indication for tonsillectomy	History of recurrent tonsillitis	Hyperplastic obstructing tonsils
Tonsil size	Small to substantially enlarged	Substantially enlarged
Tonsillar core culture test	Positive	Negative

*The tonsillar core culture test was positive if the tonsils harbored β-hemolytic streptococci or anaerobes*.

### Cell separation

Tonsils were minced in complete medium consisting of RPMI 1640 (Sigma Aldrich, St. Louis, MO, USA) supplemented with 0.3 g/l L-glutamine, 10% FCS (AH diagnostics, Aarhus, Denmark), 100 U/ml penicillin and 100 μg/ml streptomycin (Invitrogen, Carlsbad, CA, USA). The cell suspension was incubated with neuraminidase-activated sheep red blood cells (SRBC) followed by density gradient centrifugation with Ficoll-Paque (Amersham Bioscience, Uppsala, Sweden) as previously described [22]. SRBCs bind and form rosettes with human T cells through their affinity for CD2 (pre-treatment with neuraminidase increased this binding). After centrifugation on a Ficoll gradient, the non-rosetting cells are found at the interface fraction while the rosetting T cells are obtained from the pellet [22,23]. The T cells are thereafter obtained by lysing the SRBCs with dH_2_0 and 1.4 M NaCl. Different subsets of T cells were further isolated using the MACS magnetic labeling system (Miltenyi Biotec, Cologne, Germany) according to instructions of the manufacturer. Briefly, cells were incubated in 4°C with antibody-conjugated microbeads in buffer containing PBS supplemented with 0.5% FCS and 2 mM EDTA, and separated on a LS column placed on a magnetic separator. Untouched CD8^+ ^T cells were isolated by an indirect magnetic labeling system using antibodies against CD4, CD14, CD16, CD19, CD36, CD56, CD123, TCRγ/δ and Glycophorin A to deplete CD4^+ ^T cells, monocytes, granulocytes, B cells, γ/δ T cells, NK cells, DCs, and erythroid cells (CD8^+ ^T Cell Isolation Kit II, Miltenyi Biotec). Untouched CD4^+ ^T cells were isolated in a similar manner (CD4^+ ^T Cell Isolation Kit II, Miltenyi Biotech) by depletion of non-CD4^+ ^cells. The CD4^+ ^subset was occasionally further separated based on the expression of the chemoattractant receptor-homologous molecules expressed on Th2 cells (CRTH2) into CRTH2^-^ Th1 and CRTH2^+ ^Th2 cells using the anti-CRTH2 Microbead Kit (Miltenyi Biotech). For all protocols, the isolated cells had routinely a purity of > 95% as determined by FACS.

### RNA isolation and real-time RT-PCR

Freshly isolated T cells were lysed in RLT buffer (Qiagen, Hilden, Germany) supplemented with 1% 2-merkaptoethanol and stored in -80°C until use. RNA was extracted using RNeasy Mini Kit (Qiagen). The quantity and quality of the RNA concentration was determined by spectrophotometry based on the A_260_/A_280 _ratio. Omniscript Reverse Transcriptase kit (Qiagen) and oligo(dT)_15 _primer (Novagen, Nottingham, UK) were used for first-strand cDNA synthesis with an aliquot of 20 ng RNA as starting material. The obtained cDNA was diluted with water and 18 ng was used for amplification. The real-time PCR was performed on a Smart Cycler (Cepheid, Sunnyvale, CA, USA) using TaqMan Universal PCR Master Mix, No AmpErase UNG and Assay-on-Demand Gene Expression products (Applied Biosystems, Foster City, CA, USA) containing unlabeled primers and MGB probe (FAM™ dye-labeled). The thermal cycler was programmed to perform an initial set-up (95°C, 10 min) and 45 cycles of denaturation (95°C, 15 s) followed by annealing/extension (60°C, 1 min). To ensure proper function of the probes, B cells and neutrophils were used as controls. The gene expression was assessed using the comparative cycle threshold (Ct) method . The relative amounts of mRNA for the TLRs were determined by subtracting the Ct values for these genes with the Ct value for the housekeeping gene β-actin (ΔCt) [24]. The amount of mRNA is expressed in relation to 100,000 mRNA molecules of β-actin (100,000 × 2^-ΔCt^) and presented as mean values ± SEM.

### FACS analysis

Flow cytometry analyses were performed on a Coulter Epics XL flow cytometer (Beckman Coulter, Marseille, France). Live lymphocytes were gated based on forward and side scatter properties and 10,000–15,000 events were collected and analyzed using Expo32 ADC analysis software (Beckman Coulter). The following anti-human mAbs were used: CD3-FITC (UCTH1) and CD3-ECD/CD8-FITC/CD4-PE (UCTH1/B9.11/13B8.2) from Immunotech (Beckman Coulter, Marseille, France), CD8-PECy5 (RPA-T8) and CD4-PECy5 (RPA-T4) from eBioscience (San Diego, CA, USA) and TLR2-FITC (TL2.1), TLR3-PE (40C1285), TLR4-FITC (HTA125) and TLR9-FITC (26C593) from AMS Biotechnology (Abingdon, UK). Unlabeled mAbs against TLR1 (GD2.F4) and TLR5 (85B152.5) from Acris antibodies (Hiddenhausen, Germany) were used together with Alexa Fluor 488 mouse IgG1 or IgG2a labeling kit from Molecular Probes (Eugene, OR, USA). Isotype controls relevant for each Ab were used for background staining. Serial dilutions of the TLR Abs were performed to determine the optimal working titres, and as positive controls B cells, monocytes and DCs were used. For the detection of TLRs, intracellular staining of the cells was performed. Freshly isolated CD4^+ ^and CD8^+ ^cells were fixed in 4% formaldehyde and permeabilized in PBS containing 0.1% Triton × 100. Cells were either incubated for 30 min in RT with primary mAbs targeted with Alexa Fluor 488, or for 30 min in 4°C with direct-conjugated mAbs. To block unspecific binding, PBS supplemented with 2% FCS was used for all labeling and washing steps.

### Immunohistochemistry

The morphological localization of TLR proteins in tonsils was investigated using immunohistochemistry. Tissue preparations were embedded in paraffin, cut in 3 μm thick sections, mounted on glass slides and stored in -80°C until use. Prior to visualization of proteins, the sections were treated with xylene to remove the paraffin and rehydrated using ethanol. To facilitate binding of the Abs, the sections were treated with Target retrieval solution (DakoCytomation, Copenhagen, Denmark) for 20 min in a microwave oven, followed by Triton × 100 (1%) for increased membrane permeability. To quench endogenous peroxidase activity, sections were incubated in 0.03% hydrogen peroxide for 10–15 min. If needed, sections were incubated for 20 min in PBS containing 2% FCS to block non-specific binding. The following primary Abs were used, and applied to the sections for 1 h: CD4 (IF6) from Novocastra (Newcastle upon Tyne, UK), CD8 (C8/144B) and CD20cy (L26) from DakoCytomation, TLR1 (GD2.F4), TLR5 (SM7102P), TLR7 (rabbit polyclonal) and TLR9 (26C593) from Acris antibodies, as well as rabbit pAbs against TLR2 and TLR3 from AMS Biotechnology. Subsequently, HRP-labeled goat anti-mouse or goat anti-rabbit polymer was incubated with the sections for 30 min, followed by 3,3'-diaminobenzidine (DAB) or 3-amino-9 ethylkarbazole (AEC) respectively (DakoCytomation EnVision^+ ^System-HRP kits). To exclude unspecific staining of the HRP-labeled polymer, antibody diluent without primary Ab was used as negative control. At some occasions, sections were counterstained with Mayer's hematoxylin. The sections stained with AEC were mounted in Faramount aqueous mounting medium (Dako), whereas the DAB-stained sections were dehydrated with increasing concentrations of ethanol, rinsed in xylene and mounted in Pertex (Histolab). TBS (pH 7.6) supplemented with 0.05% Tween 20 was used for all washing steps.

### Statistics

Statistical analysis was performed using GraphPad Prism 4 (San Diego, CA, USA). Student's *t*-test was used to determine statistical differences for unpaired, with the Welch test if variances were non-homogenous, and paired observations. P-values ≤ 0.05 were considered statistically significant.

## Results

### Expression pattern of TLR1-TLR10 in subsets of human T cells

**Figure 1:**
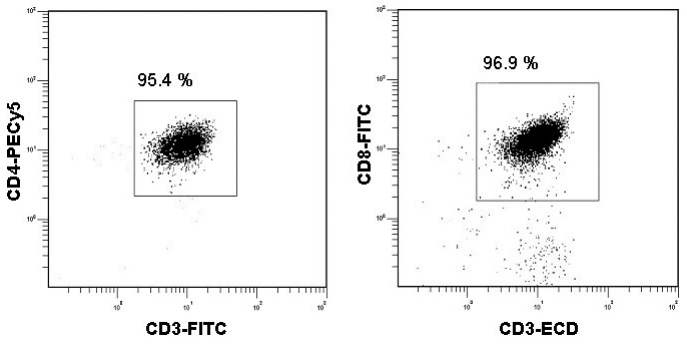
**The isolated CD4^+ ^and CD8^+ ^T cells are highly purified.** Tonsils were minced and T cells were separated from other mononuclear cells using SRBC rosetting followed by density gradient centrifugation. CD4^+ ^and CD8^+ ^T cells were further isolated by negative selection using magnetic beads. The isolation procedure was accompanied by FACS analysis of surface phenotype (purity > 95%).

**Figure 2:**
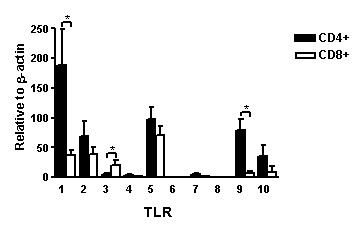
**Expression pattern of TLR transcripts in subsets of human tonsil T cells. **Freshly isolated CD4^+ ^(filled bars) and CD8^+ ^T cells (open bars) were analyzed for TLR1-TLR10 expression using quantitative real-time PCR. Data from experiments with cells from different patients are summarized (CD4^+^, n = 22, CD8^+^, n = 21), depicted in relation to the housekeeping gene****β-actin as 2**^-^**^ΔCt^× 10^5 ^and presented as mean values ± SEM (*p < 0.05).

Analysis of TLRs in T cells requires removal of contaminating cells expressing high levels of TLRs, such as monocytes, B cells and DCs. FACS analysis of the purified T cell subsets routinely showed a purity of > 95 % (Fig 1), which considerably diminished the possibility of contamination. The freshly isolated CD8^+^, CD4^+^, CD4^+^/CRTH2^- ^and CD4^+^/CRTH2^+ ^cells from human tonsils were analyzed for expression of TLR1-TLR10 transcripts using quantitative real-time RT-PCR, and all data are presented in relation to the housekeeping gene β-actin. The tonsils used were randomly selected, independent of the patients' clinical diagnosis. Hence, the cells originated from both infected and hyperplastic tonsils. T cells expressed 8 out of the 10 presently known TLRs, and among them TLR1, TLR2, TLR5, TLR9 and TLR10 were most prominent (Fig 2). Expression of TLR3, TLR4 and TLR7 was relatively low, whereas TLR6 and TLR8 were undetectable. CD8^+ ^cells generally expressed lower levels of TLR mRNA than CD4^+ ^cells. Comparison of the expression levels of single TLRs in the different cell types revealed that CD4^+ ^cells had significantly higher mRNA expression levels of TLR1 and TLR9 than CD8^+^, while in contrast TLR3 expression was significantly higher in CD8^+ ^cells. TLR4 and TLR7 levels were close to the detection limit, why no reliable statistical analyses could be made. Furthermore, no significant differences in mRNA expression were found between CD4^+^/CRTH2^- ^Th1 and CD4^+^/CRTH2^+ ^Th2 cells (n = 21; data not shown).

**Figure 3:**
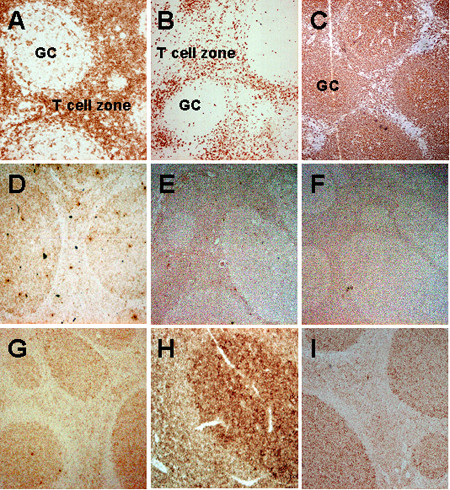
**Immunohistochemical staining of tonsillar tissue. **Tonsil sections were stained with Abs against (A) CD4 (diluted 1:400), (B) CD8 (1:50), (C) CD20 (1:1500), (D) TLR1 (1:50), (E) TLR2 (1:50), (F) TLR3 (1:50), (G) TLR5 (1:50), (H) TLR7 (1:50) and (I) TLR9 (1:50), localized using DAB or AEC, which stain tissues brown and red respectively, and analyzed by microscopy (magnification 40–100×).

To determine whether this observed mRNA expression pattern actually reflected the expression of the TLR proteins, immunohistochemistry and flow cytometry were carried out. Immunohistochemical staining of tonsils with Abs against CD4, CD8 and CD20 as well as against TLR1, TLR2, TLR3, TLR5, TLR7 and TLR9 was performed to identify the cellular location of the receptors. Ab against TLR4 was evaluated but found not to function in a satisfactory way, and no Ab against TLR10 was available on the market. As expected, CD4^+ ^cells were found both in the T cell zones and in the germinal centers (GCs) (Fig 3A) where they provide help during B cell differentiation. CD8^+ ^cells on the other hand were only detected in the T cell zones of the tonsils (Fig 3B). B cells, stained with CD20, were located mainly in the GCs (Fig 3C). TLR1, TLR5, TLR7 and TLR9 were found highly expressed within the GCs, but also in the adjacent T cell zones, whereas TLR2 and TLR3 expression was more intense in the T cell zones (Fig 3D-I). Although the staining of the TLRs does not discriminate between T cells and other TLR-expressing cells, the locations of the receptors are clearly shown. Collectively, TLR1, TLR2, TLR3, TLR5, TLR7 and TLR9 were all present in the T cell zones, but also in the GCs, of human tonsillar sections, which supports the presence of TLR mRNA presented above.

**Figure 4:**
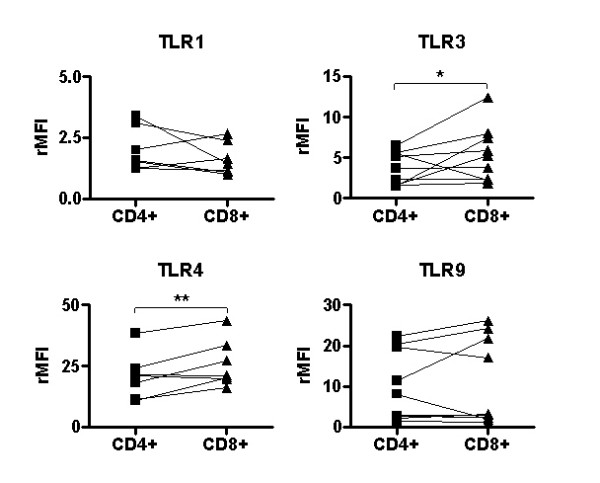
**CD8^+ ^T cells express higher levels of TLR3 and TLR4 than CD4^+ ^T cells. **Freshly isolated CD4^+ ^and CD8^+ ^T cells from human tonsils were stained intracellularly with Abs against CD4 or CD8 and TLR1, TLR3, TLR4, TLR9 or control Ab and analyzed by FACS. Data, summarized from eight independent experiments, are expressed as rMFI ± SEM (*p = 0.05, **p < 0.01).

In order to verify the presence of the TLR proteins as well as to confirm the differences observed between CD4^+ ^and CD8^+ ^cells at transcriptional level, Abs against the various TLRs were used for flow cytometry analyses. The fluorescence intensity of the TLRs varied markedly between different donors. Therefore, the TLR expression was calculated as relative mean fluorescence intensity (rMFI = TLR Ab/corresponding isotype control) [25]. CD8^+ ^T cells displayed a higher level of TLR3 than CD4^+ ^cells, which is in line with the real-time PCR results (Fig 4). The higher expression of TLR1 and TLR9 previously seen in CD4^+ ^cells could not be confirmed statistically, although there was a trend towards a higher TLR1 expression in CD4^+ ^cells. An interesting finding was that TLR4, which was poorly expressed at mRNA level, was clearly present in both CD4^+ ^and CD8^+ ^cells. In addition, the receptor expression was significantly higher in CD8^+ ^compared to CD4^+ ^cells. Moreover, no differences in expression of TLR2 and TLR5 proteins were found between the subsets (data not shown).

### Influence of chronic tonsillar infection on TLR expression

**Figure 5:**
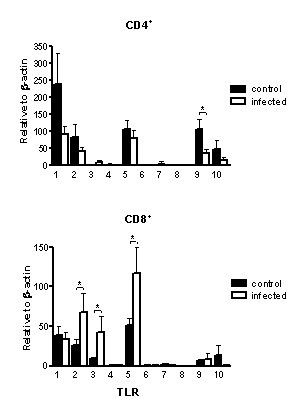
**Altered TLR expression in response to tonsillar infection. **Freshly isolated CD4^+ ^and CD8^+ ^T cells from infected tonsils (open bars) and controls (filled bars) were analyzed for TLR1-TLR10 expression using quantitative real-time PCR. Data from experiments with cells from different patients are summarized (CD4^+^, n = 14–15, CD8^+^, n = 7–13), depicted in relation to the housekeeping gene β-actin as 2**^-^**^ΔCt^× 10^5 ^and presented as mean values ± SEM (*p < 0.05).

In this study the expression profile of the 10 presently known TLRs has been characterized in freshly isolated CD4^+ ^and CD8^+ ^T cells. The results show expression of most TLRs, but also a differential expression in the two cellular subsets. Next, it has previously been reported that microbial stimuli affect the expression of the cognate TLR [26]. Therefore, we wished to investigate whether infection has an effect on the TLR expression. To address this issue, the TLR expression in cells from recurrently infected tonsils was compared with controls. In the infected tonsils several bacterial strains were found, including β-hemolytic streptococci (group A, C and G) and anaerobes. The results revealed that TLR2, TLR3 and TLR5 transcripts were up-regulated in CD8^+ ^cells from infected tonsils, whereas in CD4^+ ^cells, TLR9 transcripts were down-regulated (Fig 5).

**Figure 6:**
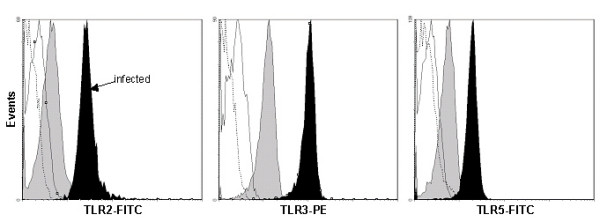
**Enhanced TLR2, TLR3 and TLR5 expression in CD8^+ ^T cells from infected tonsils (black area) compared to controls (gray area). **Freshly isolated CD8^+ ^T cells were stained intracellularly with Abs against CD8 and TLR2, TLR3 or TLR5 and analyzed by FACS. Also shown are isotype controls (infected, black line; control, dashed line). Experiments were performed with cells from four patients out of each group. One representative experiment from each group is shown.

In order to confirm the observed differences between infected tonsils and controls, freshly purified cells were analyzed for expression of TLR proteins using flow cytometry. As previously mentioned, the fluorescence intensity varied markedly between different donors, why it was impossible to conduct statistical analyses. However, we could see a clear trend that CD8^+ ^cells from infected tonsils displayed an increase in TLR2, TLR3 and TLR5 (Fig 6). In contrast, the down-regulation observed with TLR9 could not be confirmed at protein level. All in all, these observations demonstrate an altered TLR profile in patients with a history of recurrent tonsillitis caused by GAS.

## Discussion

The expression profiles as well as the functions of the TLRs have been studied extensively in various types of immune cells. However, information regarding TLRs in lymphocytes other than B cells is still limited. The present study demonstrates mRNA and corresponding proteins for TLR1-TLR10 in different subsets of T lymphocytes. Broad patterns of TLRs are found in all cellular subsets investigated and marked differences between CD4^+ ^and CD8^+ ^T cells can be noted. In addition, the TLR expression profile in cells derived from patients with chronic tonsillitis differs from the pattern found among healthy controls.

Using purified human tonsil T cells we found transcripts encoding 8 of the 10 presently known TLRs, with TLR1, TLR2, TLR5, TLR9 and TLR10 being most prominently expressed. This is in agreement with previous studies that collectively have demonstrated the presence of most TLRs in T cells derived from peripheral blood [26-29]. The most notable differences between the present and previous blood based reports [26,29] are that we found higher mRNA levels of TLR10 and no expression of TLR6 and TLR8. These discrepancies might be related to the functional and phenotypical differences between T cells from the different origins [9]. mRNA levels do not always correlate with the actual protein levels. Hence the present study extends and complements the transcriptional data, demonstrating the presence of the corresponding TLR proteins.

Most studies on TLRs have been focusing on immune cells known to be involved in the innate immunity, such as DCs, monocytes and granulocytes. Although T cells express low levels of TLRs compared to other immune cells, they do indeed express a broad TLR repertoire. Traditionally T cells are not believed to play a role in the initial phase of the innate immune response. However, the finding of TLRs in T cells might reflect a direct link to the adaptive immunity [28]. This question has been addressed in previous studies examining the effects of various TLR ligands on T cell activation. TLR2, activated by bacterial lipoproteins, has been described as a co-stimulatory receptor to human CD4^+ ^T cells [30], and flagellin and R-848 are reported to directly stimulate memory CD4^+ ^T cells via TLR5 and TLR7/8 respectively [29]. The emphasis has however been on CpG-DNA, known to provide mitogenic stimuli to human B cells and activate APCs. If the reported effects on T cells are direct or mediated via APCs is still a matter of controversy [21,31-34], and further functional studies to demonstrate the relevance of TLR stimulation are required.

The present study also demonstrates a differential expression of TLRs in CD4^+ ^and CD8^+ ^T cells. This is not surprising since their activation and effector functions are divergent. Zarember *et al *[28] have performed a study where the TLR expression in CD4^+ ^and CD8^+ ^subsets of T cells is examined using real-time PCR. Our results are consistent with theirs, demonstrating that CD4^+ ^cells express higher levels of TLR1, TLR4 and TLR9 than CD8^+ ^cells, and that CD8^+ ^express a higher level of TLR3. In addition, we found that mRNA expression levels do not always correlate with the corresponding protein levels assessed by flow cytometry. These discrepancies were most evident for TLR9. Similar differences when TLR mRNA and protein do not correlate have previously been described in human leukocytes [14,35]. A commonly used explanation for this phenomenon is that proteins might be consumed after they have been activated. Other possible explanations might be related to transcriptional control as well as mRNA and protein half-life [36,37].

An interesting finding in the present study is that CD8^+ ^cells, in comparison to CD4^+ ^cells, exhibit a higher expression of TLR3, which is involved in the recognition of dsRNA produced by viruses during their replication [13]. Upon infection with viruses, fragments of the virus are presented on MHC class I and recognized by CD8^+ ^cells, resulting in the induction of a cytotoxic T cell response [38,39]. It is tempting to speculate in whether TLR3 is yet another way for CD8^+ ^T cells to recognize viral infections, and thus have a role in activation of the cytotoxic T cell response.

Microbial stimulation is known to alter the TLR expression in various cell types [27,28,40]. However, no such information has been presented for T cells. Therefore we decided to compare the TLR expression in T cells from recurrently infected tonsils with non-infected/hyperplastic tonsils. The hyperplastic tonsils were culture-negative and can therefore function as appropriate controls in relation to the infected tonsils. This assumption is also supported by the fact that hyperplastic tonsils are a relatively common finding also among normal healthy children not undergoing tonsillectomy. Nonetheless, it is important to bear in mind that these "control tonsils" might not be fully healthy tissues. Therefore, it cannot be completely ruled out that there is some immunological relevant cause of the hyperplasia that effects the TLR expression on those tissue samples. Even so, we made the observation that CD8^+ ^T cells from infected tonsils express a higher level of TLR2, TLR3 and TLR5. In contrast, TLR9 was found down-regulated in CD4^+ ^T cells during tonsillar infection. The latter finding could however not be confirmed at protein level.

One explanation for the infection-dependent induction of TLR2 might be related to the presence of Gram-positive *Streptococcus pyogenes*, such as GAS. TLR2 is known to be activated by structures on Gram-positive bacteria such as lipoproteins and peptidoglycan [16], but the receptor has also been described to recognize a soluble factor from group B streptococci isolated from newborn infants with sepsis [41]. If streptococci found in upper airway infections secrete a similar factor that can be detected by TLR2 has not yet been addressed, but if so, might explain the induction of TLR2. Infected tonsils often concomitantly harbor bacteria and viruses [4]. dsRNA from the latter might be of special importance for the activation of TLR3, whereas flagellated bacteria might have contributed to the enhanced expression of TLR5. The literature on how the TLR expression is affected by microbial stimuli is partly contradictory. Hornung *et al *[26] demonstrated a decrease in TLR9 in plasmacytoid dendritic cells (PDC) and B cells in response to CpG-DNA stimulation, whereas Muzio *et al *[27] found an increase in TLR4 in polymorphonuclear cells (PMN) and monocytes, as well as in TLR2 in PMN upon exposure to their cognate PAMPs. Furthermore, an inflammation-dependent induction in TLR2 and TLR4 expression in intestinal macrophages has been demonstrated [40], as well as both a positive and negative regulation of these receptors in granulocytes and monocytes after incubation with *E. coli *[28]. Taken together, it is evident that the TLR expression is affected by microbial stimulation, albeit no general rule appears to apply. If the TLR expression becomes positively or negatively regulated seems to be related to the cell types involved as well as to variations in the external milieu [27]. Further, the well-defined patterns of TLRs in different cell types imply that each cell type has a certain way to respond to microbial stimulation [28].

## Conclusion

We have demonstrated the presence of a broad repertoire of TLRs in T cells, a differential expression in CD4^+ ^and CD8^+ ^T cells, along with an altered TLR expression in response to tonsillar infection caused by *Streptococcus pyogenes *or anaerobes. The expression of TLRs in T cells further stresses the biological role of TLRs in the adaptive immune response, and raises questions regarding the role of TLRs in T cell activation both with and without APCs. Thus, further functional studies of the relevance of TLR stimulation on T cells are of great importance. Moreover, the infection-dependent alterations in TLR expression in T cells support the idea that TLRs are important players in immune reactions against infection.

## Abbreviations

TLR: Toll-like receptor

PAMP: pathogen-associated molecular pattern

GAS: group A β-hemolytic streptococci

Th1: type 1 T helper cell

Th2: type 2 T helper cell

CRTH2: chemoattractant receptor-homologous molecules expressed on Th2 cells

CTL: cytotoxic T lymphocyte

APC: antigen-presenting cell

## Competing interests

The author(s) declare that they have no competing interests.

## Authors' contributions

AM performed the experimental work, analyses and interpretation of data, drafted the manuscript and was involved in the design of the study. MA and LOC made substantial contributions to conception and design of the study, and were involved in critically revising the manuscript. All authors read and approved the final manuscript.
